# Maritime Spectrum Sensing Based on Cyclostationary Features and Convolutional Neural Networks

**DOI:** 10.3390/e27080809

**Published:** 2025-07-28

**Authors:** Xuan Geng, Boyu Hu

**Affiliations:** College of Information Engineering, Shanghai Maritime University, Shanghai 201306, China; 19121695639@163.com

**Keywords:** MCRN, spectrum sensing, cyclostationary feature, CNN, Transfer Learning (TL)

## Abstract

For maritime cognitive radio networks (MCRN), spectrum sensing (SS) is challenging due to the movement of the sea, channel interference, and unstable link quality. Besides the basic sensing capabilities that are required, SS in MCRN also requires the ability to adapt to complex and dynamic environments. By transforming spectrum sensing into a classification problem and leveraging cyclostationary features and Convolutional Neural Networks (CNN), This paper proposes a classification-guided TC2NND (Transfer Cyclostationary- feature and Convolutional Neural Networks Detection) spectrum sensing algorithm, which regards the maritime spectrum sensing as a classification problem. The TC2NND algorithm first classifies the received signal features by extracting cycle power spectrum (CPS) features using the FFT (Fast Fourier Transform) Accumulation Method (FAM), and then makes a judgment using a variety of C2NND decision models. The experimental results demonstrate that the proposed TC2NND algorithm could achieve a detection probability of 91.5% with a false-alarm probability of 5% at SNR = −10 dB, which significantly outperforms the conventional methods.

## 1. Introduction

Cognitive radio (CR) addresses the challenge of uneven spectrum resource utilization by employing dynamic spectrum allocation strategies. This allows secondary users (SUs) to access frequency bands used by primary users (PUs) without disrupting their communications, thereby offering a promising solution to meet growing bandwidth demands in a resource-limited spectrum environment. Spectrum sensing (SS) is a critical component for enabling CR technology. In SS, SUs must promptly detect the presence or absence of PUs to quickly release frequency resources when PUs appear, ensuring uninterrupted transmission for PUs. Therefore, we need SS to have high accuracy, real-time performance, and low complexity.

The traditional spectrum sensing algorithms include energy detection [1], matched filter detection [2], and cyclostationary feature detection [3]. These algorithms have different implementation principles and, as a result, exhibit distinct advantages and disadvantages in terms of detection performance. The propagation of radio waves in water is subject to variation, and the unevenness of the sea surface can result in the occurrence of negative interference in the reflection path [4]. The complexity and variability of the maritime environment make maritime spectrum sensing a challenge with high uncertainty and dynamic changes. In the past, researchers have typically taken the Cooperative Spectrum Sensing (CSS) approach. For instance, one study [5] proposed an entropy-based CSS method that demonstrated improved detection rates and stability compared to energy detection. Another approach [6] introduced a biologically inspired CSS algorithm, which selects SUs for collaboration based on the sea state to achieve energy-efficient, highly adaptive spectrum sensing with a robust detection probability. Additionally, a centralized double-threshold CSS scheme for maritime communication networks was proposed in [7], which involved consolidating the received signal energy data from each cognitive node at a fusion center for decision-making.

Despite their effectiveness, traditional spectrum sensing methods are often limited by their sensitivity to noise and reliance on prior knowledge of PU signals. The rapid advancement of deep-learning technologies presents a compelling alternative to address these limitations, offering enhanced learning capabilities, automatic feature extraction, and robust generalization [8]. For example, in [9], the author uses LSTM (Long Short-Term Memory) for spectrum sensing to identify the presence or absence of a Primary User (PU) by learning the temporal correlation in the spectrum data. In [10], a Convolutional Neural Network (CNN) was employed to analyze signal spectrum maps as input, while [11] proposed a cooperative CSS approach using graph convolutional networks (GCNs), transforming the signal perception matrix into graph data suitable for analysis. The CNN-LSTM spectrum sensing model introduced in [12] leverages the covariance matrix of signals, using CNN for energy feature extraction and LSTM to learn PU activity patterns.

While existing deep learning-based spectrum sensing approaches have mainly focused on enhancing detection accuracy, they frequently neglect the crucial necessity of maintaining low false-alarm rates. This is a pivotal consideration in maritime environments where uninterrupted communication for primary users (PUs) is extremely important. To address this challenge, we propose the classification-guided multi-network TC2NND method. This approach considers the spectrum sensing problem as a classification task. We apply the FFT Accumulation Method (FAM) algorithm to estimate the CPS, which serves as input for TC2NND. The TC2NND first classifies the input based on different signal-to-noise ratio (SNR) levels, and subsequently employs multiple parallel decision models C2NND to make decisions based on the classification results. Our simulation results indicate that using a single C2NND decision model outperforms traditional energy detection and cyclostationary detection methods; however, it cannot guarantee low false-alarm rates under certain conditions. The TC2NND method effectively addresses this limitation, enhancing detection performance at low SNRs while maintaining a false-alarm rate below 1%.

## 2. System Model

### 2.1. Maritime Congnitive Radio Network

**Figure 1:**
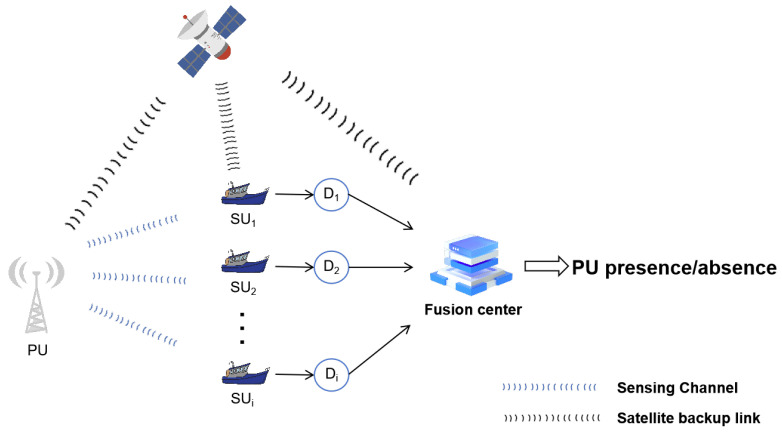
Maritime cognitive radio network.

The Maritime Cognitive Radio Network (MCRN) is illustrated in Figure 1. In this model, a PU is represented by a maritime two-way radio system capable of both transmitting and receiving signals. The fusion center, which may be located on the coast or an island, acts as a centralized node responsible for signal processing and decision-making within the network. Cognitive nodes, known as SUs, typically take the form of mobile maritime entities, such as unlicensed ships. Each vessel is equipped with devices that perform cognitive radio operations, programmed to periodically sense the radio environment to identify spectrum resources that are not currently in use by PUs. The fusion center aggregates the sensing results from cognitive node and makes a global decision regarding spectrum availability. These decision outcomes are then communicated back to the SUs. Additionally, when vessels are far from land and unable to access the fusion center directly, satellite links provide an alternative channel for connectivity to the fusion center.

### 2.2. Spectrum Sensing

Cognitive radio nodes equipped with sensing capabilities utilize antennas to actively monitor the radio frequency spectrum. By analyzing the statistical characteristics of the received signals, these nodes can discern the occupancy status of frequency bands allocated to PUs. Mathematically, the SS process can be modeled as a binary detection problem represented by the following:

(1) $$r\left(t\right)=\left\{\begin{array}{cc} n(t), & H_{0}\\ s(t)+n(t), & H_{1} \end{array}\right.$$

In this model, r(t) represents the received signal. The signal component within the received signal is defined as s(t)=h×x(t), where x(t) is the transmitted signal and *h* is the channel gain. n(t) denotes the additive white Gaussian noise (AWGN). The hypotheses $H_{0}$ and $H_{1}$ indicate the absence and presence of the PU, respectively.

The detection strategy is to compare the detection statistic ξ with a threshold ζ. The probabilities of detection ($P_{D}$) and false alarm ($P_{FA}$) are defined as follows:

(2) $$\begin{array}{c} P_{D}=P_{r}\left[\xi \geq \zeta |H_{1}\right]=P_{r}\left[H_{1}|H_{1}\right] \end{array}$$

(3) $$\begin{array}{c} P_{FA}=P_{r}\left[\xi \geq \zeta |H_{0}\right]=P_{r}\left[H_{1}|H_{0}\right] \end{array}$$

### 2.3. Channel Model

In free space, the received signal power $P_{r}$ at a distance *R* from the transmitting antenna is

(4) $$\begin{array}{c} P_{r}={\left(\frac{\lambda }{4\pi R}\right)}^{2}G_{r}P_{t}G_{t} \end{array}$$

where $P_{t}$ is the transmitting power. The $G_{t}$ and $G_{r}$ are the gain of the transmitting antenna and receiving antenna, respectively. The λ denotes the wavelength.

For maritime environments, the received signal power is affected by multi-path effects caused by sea surface reflection. The reflection includes direct reflection, the specular reflection, and the diffuse reflection [13]. The diffuse reflection signal has a low correlation with the transmitting signal and is usually considered to be a random signal. Therefore, the received signal is mainly composed of direct and specular reflection signals, whose power can be expressed as [14]

(5) $$P_{r}={\left(\frac{\lambda }{4\pi R}\right)}^{2}P_{t}G_{r}G_{t}{\left|1+\sum_{i=1}^{N}\rho_{i}e^{j\varphi_{i}}\right|}^{2}$$

where $\rho_{i}$ is the specular reflection coefficient of the *i*th reflection path. The $\varphi_{i}$ is the phase difference of the direct path with respect to the reflected path. The *N* is the number of effective reflection points. Ignoring the receiving and transmitting antenna gains, the multi-path channel gain *h* (dB) of any path from transmitter to receiver can be expressed as

(6) $$\begin{array}{cc} h & =10\log_{10}\frac{P_{t}}{P_{r}}=10\mathrm{lg}\left(\frac{{\left(\frac{4\pi Rf}{3\;\times \;10^{8}}\right)}^{2}}{{|1+\sum_{i=1}^{N}\rho_{i}e^{j\varphi_{i}}|}^{2}}\right)\\ & =-147.56+20\mathrm{lg}R+20\mathrm{lg}f-20\mathrm{lg}|1+\sum_{i=1}^{N}\rho_{i}e^{j\varphi_{i}}| \end{array}$$

where *f* is radio frequency.

## 3. Cyclostationary Feature Analysis and Dataset Generation

In a maritime cognitive wireless communication system, the PU is typically a maritime voice intercom system, primarily using frequency modulation (FM).Therefore, the signals exhibit second-order cyclostationarity, making cyclostationary analysis a valuable approach for detection.

For the transmitting signal s(t) with second-order cyclostationarity, the Fourier series expansion of its periodic Autocorrelation Function (ACF) is expressed as follows:

(7) $$R_{s}\left(t,\tau \right)=\sum_{\alpha =-\infty }^{\infty }R_{s}^{\alpha }\left(\tau \right)e^{2\pi j\alpha t}$$

where the periodic autocorrelation $R_{s}^{\alpha }\left(\tau \right)$ is defined as follows:

(8) $$R_{s}^{\alpha }\left(\tau \right)=\lim_{T\to \infty }\frac{1}{T}\int_{T}s\left(t+\frac{\tau }{2}\right)s^{*}\left(t-\frac{\tau }{2}\right)e^{-2\pi j\alpha t}dt$$

where τ is the time delay related to ACF. The *T* and α denotes period and cycle frequency, respectively.

Using the Wiener relation, the CPS can be defined as follows:

(9) $$S_{s}^{\alpha }\left(f\right)=\int_{-\infty }^{\infty }R_{s}^{\alpha }\left(\tau \right)e^{-j2\pi f\tau }d\tau$$

Based on the formulation provided in Equation (1), the CPS of received signal is denoted by

(10) $$S_{r}^{\alpha }\left(f\right)=\left\{\begin{array}{cc} S_{n}^{\alpha }\left(f\right) & H_{0}\\ S_{s}^{\alpha }\left(f\right)+S_{n}^{\alpha }\left(f\right) & H_{1} \end{array}\right.$$

where $S_{r}^{\alpha }\left(f\right)$ and $S_{n}^{\alpha }\left(f\right)$ represent the CPS of the received signal and AWGN, respectively. The $S_{s}^{\alpha }\left(f\right)$ indicates the CPS of the PU signal component. As n(t) is not a cyclostationary process, its CPS equals zero at α=0.

For the received signal r(t), its periodic ACF is computed by

(11) $$R_{r}^{\alpha }\left(\tau \right)=\lim_{T\to \infty }\frac{1}{T}\int_{T}u\left(t+\frac{\tau }{2}\right)u^{*}\left(t-\frac{\tau }{2}\right)dt$$

where

(12) $$u\left(t\right)=r\left(t\right)e^{-j\pi \alpha t}$$

According to cross-spectral analysis, we obtain

(13) $$S_{r}^{\alpha }\left(f\right)=\lim_{\Delta t\to \infty }\lim_{T\to \infty }\frac{1}{T}\frac{1}{\Delta t}\int_{-\frac{\Delta t}{2}}^{\frac{\Delta t}{2}}R_{T}\left(t,f+\frac{\alpha }{2}\right){R_{T}}^{*}\left(t,f-\frac{\alpha }{2}\right)dt$$

where

(14) $$R_{T}\left(t,f\right)=\int_{t-\frac{T_{0}}{2}}^{t+\frac{T_{0}}{2}}r\left(u\right)e^{-j2\pi fu}du$$

However, $S_{r}^{\alpha }\left(f\right)$ cannot be used directly as an estimate of the cyclic spectral density due to its large variance. To achieve a more accurate estimate, we need to use a smoothing technique. In this study, we utilized the FFT Accumulation Method (FAM) to smooth the CPS, thus achieving an acceptable equilibrium between cycle aliasing, computational efficiency, and cycle leakage. Compared to other algorithms (such as the periodogram method, the indirect method, and the SSCA algorithm), the FAM algorithm demonstrates better performance in terms of computational efficiency, real-time capability, and robustness under low signal-to-noise ratio conditions.

We discrete the received signal r(t) to be r(n), and the CPS estimation is obtained by

(15) $$S_{r}^{\alpha_{0}+q\Delta \alpha }\left(nL,f\right)=\frac{1}{P}\sum_{r=0}^{P-1}\frac{1}{N_{p}}R_{T}\left(rL,f+\frac{\alpha }{2}\right){R_{T}}^{*}\left(rL,f-\frac{\alpha }{2}\right)e^{-j2\pi r\frac{q}{P}}$$

where the *P* is the number of blocks that r(n) is divided into, and $N_{P}$ denotes the total number of points in each block. The value of *L* is $\frac{N_{P}}{4}$, and $q=-\frac{P}{2},-\frac{P}{2}+1,\ldots ,\frac{P}{2}-1$, and 

(16) $$R_{T}\left(n,f\right)=\sum_{n=-\frac{N_{P}}{2}}^{\frac{N_{P}}{2}-1}\omega \left(k\right)r\left(n-k\right)e^{-j2\pi f\left(n-k\right)T_{s}}$$

where $T_{s}$ is the sampling period, and ω(k) is a kaiser window with a width of $T_{\omega }=N_{p}T_{s}$. The $N_{P}$ and *P* are calculated by

(17) $$\begin{array}{c} N_{p}=2^{\left\lfloor \log_{2}\left(\frac{f_{s}}{dv}-1\right)+1\right\rfloor } \end{array}$$

(18) $$\begin{array}{c} P=2^{\left\lfloor \log_{2}\left(\frac{f_{s}}{Ld\alpha }-1\right)+1\right\rfloor } \end{array}$$

where $f_{s}$ is the sampling frequency, and dv is the desired frequency resolution, and $d\alpha =\frac{1}{dt}$ is the desired cycle frequency resolution.

**Figure 2:**
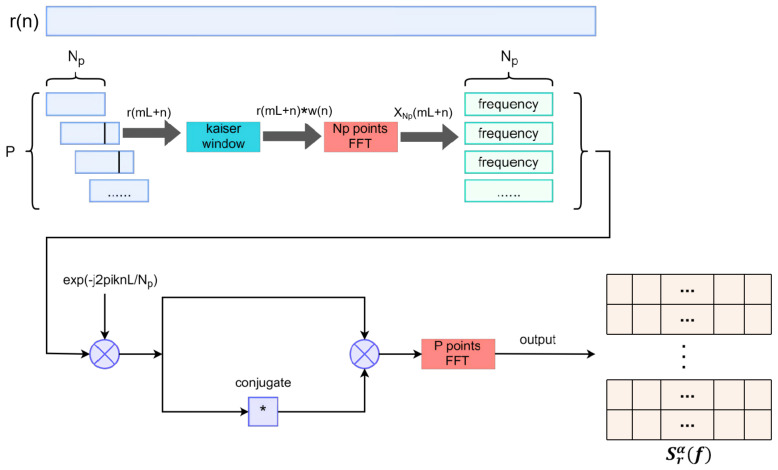
FAM algorithm.

The Block Diagram of FAM algorithm is shown in Figure 2.

Based on the CPS analysis of the signals, we generate our dataset. We use the Binary Phase Shift Keying (BPSK) modulation to generate the dataset due to robust performance of BPSK under low signal-to-noise ratio (SNR) conditions and its cyclostationary characteristics, which are well-suited to the spectrum sensing approach based on cyclostationarity in our work. First, BPSK signals $\tilde{x}\left(t\right)$ are generated and added by AWGN $\tilde{n}\left(t\right)$, in which the $\tilde{n}\left(t\right)$ may be thermal noise from the thermal motion of electronic devices, or noise caused by electromagnetic radiation. The signal x(t) passes through the maritime channel model with AWGN to obtain r(t). Subsequently, we calculated and normalized the CPS of received signal and noise, and then assigned their corresponding labels. The detailed process of dataset generation is summarized in Algorithm 1, in which $N_{s}$ is the amount of sample data generated at each Signal-to-Noise Ratio (SNR).
**Algorithm 1** Dataset Generation1:**for** snr **do**2:   **for** $n_{s}$ **do**3:     Genrate BPSK signal $\tilde{x}\left(t\right)$ with length $L_{N}$4:     $x\left(t\right)=\tilde{x}\left(t\right)+\tilde{n}\left(t\right)$5:     s(t)=h×x(t)6:     r(t)=s(t)+n(t)7:     Refer to Equation (15) to calculate and normalize the CPS of r(t), assign label = 18:     Generate noise n(t) with length $L_{N}$9:     Refer to Equation (15) to calculate and normalize the CPS of n(t), assign label = 010:   **end for**11:**end for**

**Figure 3:**
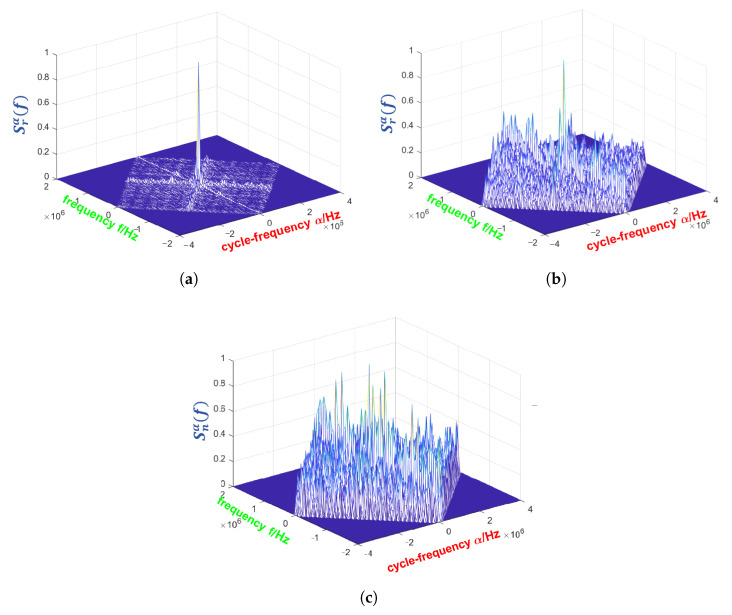
The CPS of noise and signals at different SNR. (**a**) Signal at SNR = 5 dB. (**b**) Signal at SNR = −10 dB. (**c**) Noise signal.

The cyclostationary features of the PU signal in different SNR are shown in Figure 3 by simulation, in which the CPS are calculated with FAM algorithm. As the SNR decreases, it becomes more difficult for the receiver to identify signal features and thus determine whether the PU is present.

## 4. TC2NND Model

In this section, we introduce our proposed classification-guided multi-network spectrum sensing algorithm, that is TC2NND. We will first present the C2NND and SNR classification model, which form the foundation of TC2NND. Next, we will describe the TC2NND model. Finally, we will outline the processes for offline training and online detection using TC2NND.

### 4.1. C2NND and SNR Classification Model

**Figure 4:**
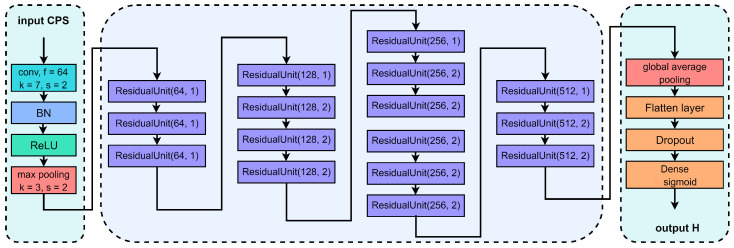
C2NND.

The previous work, where SS was based on CNN, has two significant problems. The first is that if the number of convolutional layers is too small, the network will not be able to effectively extract signal features and capture deep insights of PU’s characteristics. On the other hand, if the number of convolution layers is too large, the gradient disappearance will be caused during the training. Therefore, we integrate a residual unit in the CNN network and increase the convolutional layers, which is able to extract more complex features, as well as solves the problem of gradient disapperance by residual connections. Utilizing this improved CNN model, we named our detection model as Cyclostationary-feature and Convolutional Neural Networks Detection (C2NND), which takes the CPS of the received signal as the input of the CNN and detects the presence of the PU as the output, as shown in Figure 4.

**Figure 5:**
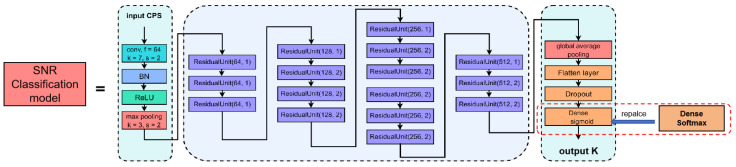
SNR classification model.

Since C2NND is designed as a binary classification model, it features a single neuron in the output layer with an Sigmoid activation function. To classify input signal features into *U* classes (U>2) to obtain the SNR classification model, we modify the activation function of the output layer to Softmax and adjust the loss function to categorical cross-entropy. This modification yields the SNR classification model, which is illustrated in Figure 5.

### 4.2. TC2NND

The CPS of the PU with low SNR will be overwhelmed by noise, resulting in overfitting of the training model and poor generalization performance at low SNR. To solve this problem, we propose to use transfer learning to transfer the weight parameters of the model with high SNR to low SNR by using the correlation of the PU’s signal, so as to improve the generalization performance and reduce the training time.

**Figure 6:**
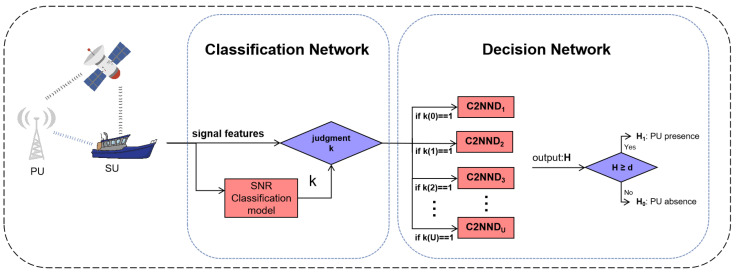
TC2NND model.

Based on the C2NND and SNR classification model mentioned above, we propose a classification-guided multi-network TC2NND model, illustrated in Figure 6. In this model, the sensed signals by SU are input into the SNR classification model to obtain *U* SNR classification. For each SNR class, the corresponding CPS of signal is input into the respective C2NND_*u*_ model to obtain *H*. By comparing the *H* with a threshold, we can obtain whether there is a PU. The proposed TC2NND includes offline training and online detection, as explained below.

#### 4.2.1. Offline Training of TC2NND

**Figure 7:**
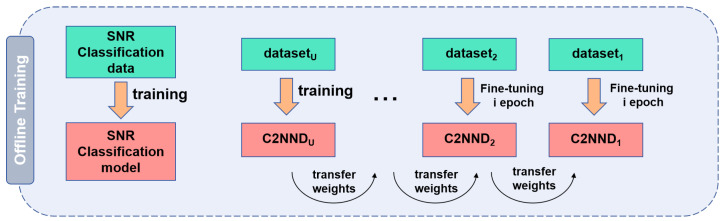
Offline training.

We need to train SNR Classification model and the Decision Network C2NND_*u*_ (u=1,2,3,…,U) in the offline training stage, as is shown in Figure 7. For training the C2NND, we employ a transfer learning strategy. First, we train C2NND_*U*_ to obtain network weights. Thereafter these weights becomes a basis for training C2NND_*U*-1_. Following this, we fine-tune the weights for *i* rounds to obtain the trained weights of C2NND_*U*-1_. This process continues as we transfer the weights to C2NND_*U*-2_ for training, ultimately completing with C2NND_1_. Thus, we complete the training for the entire network from C2NND_*U*_ to C2NND_1_.

All data generated is based on the algorithm outlined in Algorithm 1 and can be represented as follows:

(19) $$\begin{array}{c} \left(X,Y\right)=\{\left(x^{\left(1\right)},y^{\left(1\right)}\right),\left(x^{\left(2\right)},y^{\left(2\right)}\right),\cdot \cdot \cdot ,\left(x^{\left(V\right)},y^{\left(V\right)}\right)\} \end{array}$$

Here, $\left(x^{\left(v\right)},y^{\left(v\right)}\right)$ denotes the *v*-th (v=1,2,…,V) sample of the training set (X,Y). For a single example (x,y), *x* represents the input data for the neural network, in this paper is CPS, as shown in Figure 2. This CPS is structured as a two-dimensional matrix of size $\left(N_{p}+1,2P+1\right)$. Term *y* represents the label and for SNR classification data, and it can be encoded as a *U*-dimensional one-heat vector:

$$y=\left\{\begin{array}{cc} {\left[1,0,\ldots ,0\right]}^{T}, & \mathrm{go}\;\mathrm{into}\;C2NND_{1}\\ {\left[0,1,\ldots ,0\right]}^{T}, & \mathrm{go}\;\mathrm{into}\;C2NND_{2}\\ \vdots & \vdots \\ {\left[0,0,\ldots ,1\right]}^{T}, & \mathrm{go}\;\mathrm{into}\;C2NND_{U} \end{array}\right.$$

For dataset_u_, *y* can be encoded as a one-dimensional one-heat vector:

(20) $$\begin{array}{c} y=\left\{\begin{array}{c} \left[1\right],\;H_{1}\\ \left[0\right],\;H_{0} \end{array}\right. \end{array}$$

The dimension of output in each network is the same as the dimension of the input data labels. For the SNR classification model, the output is $\left[p_{0},p_{1},p_{2},\ldots ,p_{U-1}\right]$, where $p_{0}+p_{1}+p_{2}+\ldots +p_{U-1}=1$. For C2NND_*u*_, the output is [p], where *p* is the probability of predicting $H_{1}$ and (1−p) is the probability of predicting $H_{0}$.

The model is trained by minimizing the cross-entropy loss function, which is as follows:

(21) $$\begin{array}{c} \min_{\theta }\frac{1}{M}\sum_{v=1}^{M}L\left(y^{\left(v\right)},f\left(x^{\left(v\right)};\theta \right)\right)=\min_{\theta }\left\{-\frac{1}{M}\sum_{v=1}^{M}\sum_{j=1}^{S}y_{j}^{\left(v\right)}\log\left(f_{j}\left(x^{\left(v\right)};\theta \right)\right)\right\} \end{array}$$

Here, *M* denotes the number of samples entered into the network at a time, $y^{\left(v\right)}$ denotes the labeled value of the *v*-th sample, θ denotes the network parameters, and $f(x^{\left(v\right)};\theta )$ denotes the predicted value of the *v*-th sample.

We use the Adam optimizer for gradient descent to find the optimal model parameters θ that minimize the loss function. Once the training epoch has reached a predetermined value or the loss function has reached a stable point, the training process is complete. Subsequently, the model with the lowest loss can be utilized for online detection.

#### 4.2.2. Online Detection of TC2NND

After obtaining the well-trained TC2NND network through offline training, we perform online detection, which is described in Algorithm 2.
**Algorithm 2** Online detection of TC2NND  1:A/D sampling of r(t) to get r(n)  2:Refer to Equation (15) to calculate the CPS of r(n)  3:**for** each element *u* in *k* **do**  4:   **if** k(u)==1 **then**  5:     Select Decision model ${\mathrm{C2NND}}_{u}$  6:     Input CPS of r(n) into ${\mathrm{C2NND}}_{u}$ to get output *H*  7:     **if** $H\geq d_{u}$ **then**  8:        PU exists  9:     **else**10:        PU does not exist11:     **end if**12:   **end if**13:**end for**

## 5. Simulation Results

### 5.1. Simulation Environment

In this study, the number of PU and SU in the simulation is set to one, thereby indicating that the system is operating in single-user spectrum sensing mode. The simulation parameters are shown in the Table 1.

### 5.2. Performance of TC2NND

**Figure 8:**
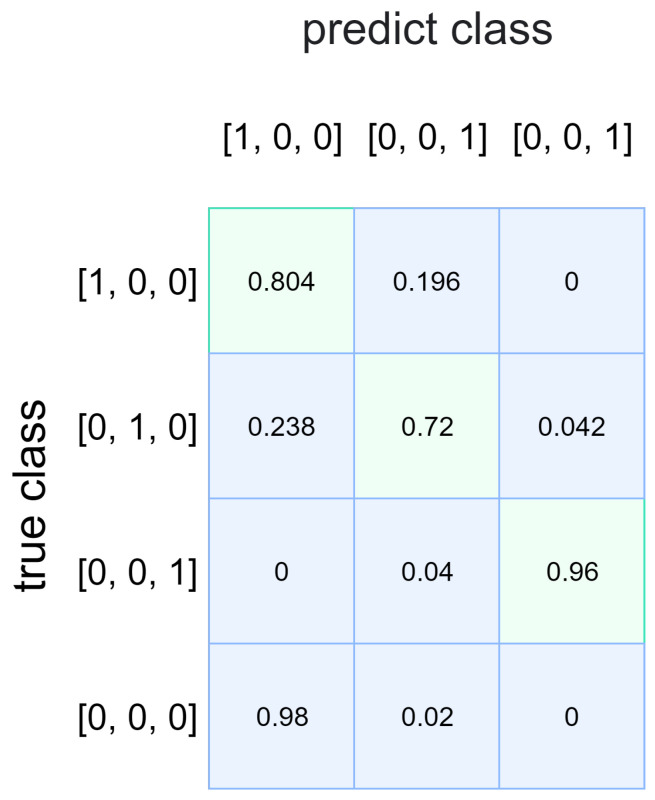
The performance of the SNR classification model.

In order to validate the performance of TC2NND proposed in this paper, a comparison is made with other methods, including energy detection, cyclostationary feature detection, and a single detection model, C2NND. First, we analyze the performance of the SNR classification model, as illustrated in Figure 8. The value [0, 0, 0] represents the noise signal. Notwithstanding the absence of noise signals in the training data, the classifier exhibits a proclivity to categorize these signals as PU signals with low SNR. Specifically, 98% of noise signals are classified as PU signals in the [1, 0, 0] category, while 2% are identified as PU signals in the [0, 1, 0] category. Consequently, the SS detection model corresponding to the identified category is utilized to minimize the impact of noise on the decision-making process. The results indicate that PU signals in the [0, 0, 1] category can be accurately estimated; however, there is a slight mutual misclassification observed between PU signals in the [1, 0, 0] and [0, 1, 0] categories. The misclassification at low SNR mainly result from the similarity between the CPS of noise and that of the signal. However, the subsequent decision model, ${\mathrm{C2NND}}_{1}$, is able to correct the misclassification of signals under low SNR conditions.

**Figure 9:**
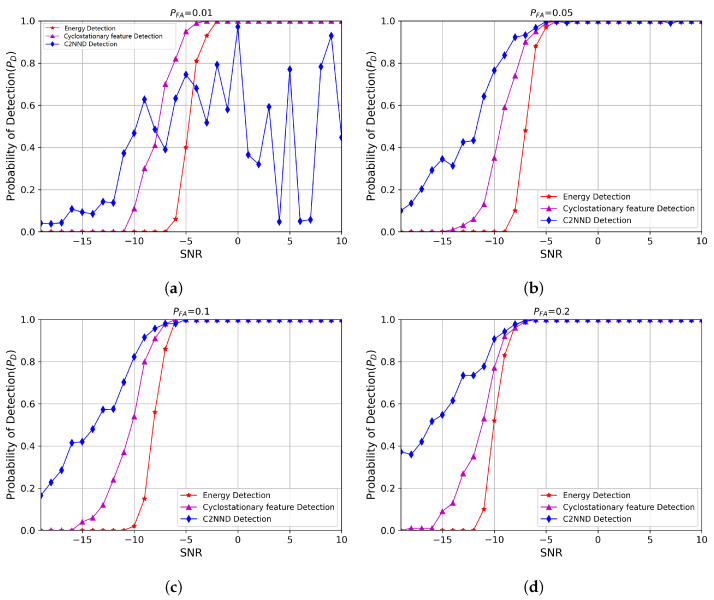
Probability of detection of C2NND at different $P_{FA}$. (**a**) $P_{FA}=0.01$. (**b**) $P_{FA}=0.05$. (**c**) $P_{FA}=0.1$. (**d**) $P_{FA}=0.2$.

Next, we verified the performance of using C2NND. by comparing it with the energy detection and cyclostationary feature detection methods, as shown in Figure 9. It can be observed that the C2NND exhibits enhanced detection performance when $P_{FA}>$ 0.01; however, its detection is unreliable when $P_{FA}=$ 0.01. This indicates that relying solely on a C2NND in complex maritime environments may not be sufficient to achieve optimal detection performance at very low $P_{FA}$.

**Figure 10:**
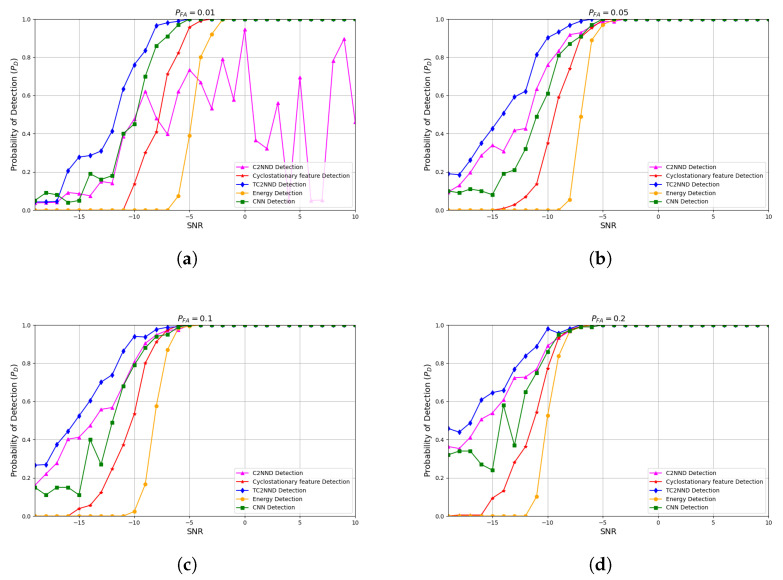
Probability of detection of TC2NND at different $P_{FA}$. (**a**) $P_{FA}=0.01$. (**b**) $P_{FA}=0.05$. (**c**) $P_{FA}=0.1$. (**d**) $P_{FA}=0.2$.

To determine whether the proposed TC2NND effectively addresses this issue, further comparative analyses are presented in Figure 10. It can be seen from the comparison results that the TC2NND SS algorithm solves the problem that the C2NND SS algorithm cannot guarantee a very low $P_{FA}$. Additionally, under the same circumstances, the TC2NND SS algorithm has slightly better detection performance than the C2NND. For example, when satisfying $P_{FA}$ = 0.2, the TC2NND detection probability can reach 85% at SNR = −12 dB, which is better than 75% for C2NND and 38% for cyclostationary feature detection. In particular, when $P_{FA}$ = 0.01 is satisfied, T2CNND achieves stable detection, and the detection probability of TC2NND can be up to 78% under the condition of SNR = −10 dB, which is a 65% improvement over the cyclostationary feature detection. This improvement can be attributed to the fact that the sub-detection models in the TC2NND algorithm are focused on learning the characteristics of PU signals within their respective categories, enabling targeted learning that enhances detection performance. In addition, the TC2NND method also outperforms the CNN-based training approach, primarily because the latter does not include the SNR-based preprocessing before training. In summary, the TC2NND method provides more stable detection in complex maritime environments without interfering with PU, while also improving detection rates compared to traditional methods.

Besides the comparison of the probability of detection, we also compared the computational complexity with total floating-point operations (FLOPs) and the average processing delay of different methods. The results are presented in Table 2. Although the neural network-based learning methods introduce higher computational complexity compared to traditional approaches, the increase in complexity is not exponential and remains within an acceptable range. The processing time can be reduced by incorporating hardware accelerators.

In order to verify the role of the Transfer Learning (TL) technology applied in the TC2NND SS algorithm, a comparative experiment between the TC2NND SS model and the TC2NND SS model without TL technology was set up, including the training cost and detection performance of each sub-detection model. Table 3 presents the training time, validation loss, and accuracy of each of the three C2NND decision models used in the proposed TC2NND method based on transfer learning. Table 4 and Table 5 show the corresponding loss and accuracy results when transfer learning is not applied to TC2NND, with the training time for each of the three C2NND sub-models doubled and tripled, respectively.

According to the comparison between Table 3 and Table 4, it can be seen that at a similar level of training time, the sub-decision model in TC2NND has lower loss and higher accuracy on the validset. According to the comparison between Table 3 and Table 5, it can be seen that at the level with twice the training time of TC2NND, the performance of each sub-decision model in TC2NND without TL technology is slightly improved on the validset, but its loss and accuracy is still inferior to the TC2NND model with TL technology applied. This shows the role of TL technology in TC2NND.

**Figure 11:**
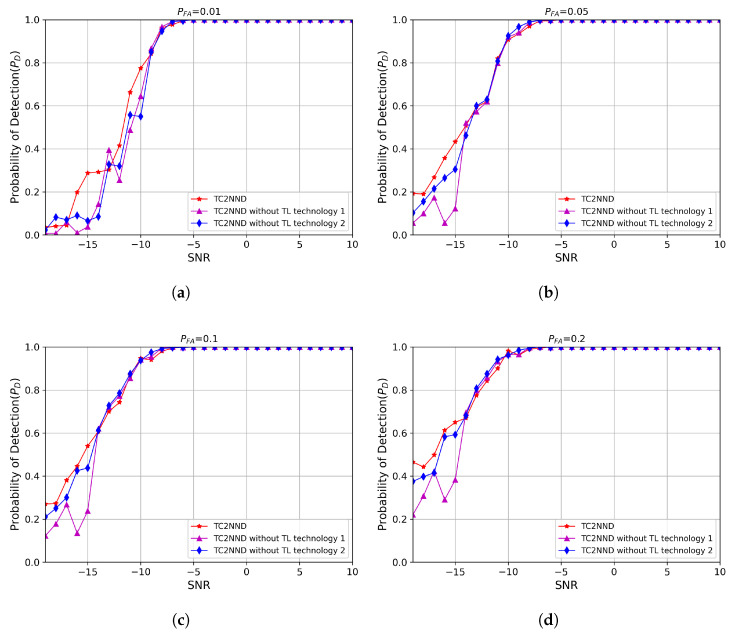
Probability of detection of TC2NND with or without TL technology. (**a**) $P_{FA}=0.01$. (**b**) $P_{FA}=0.05$. (**c**) $P_{FA}=0.1$. (**d**) $P_{FA}=0.2$.

The detection performance of the models corresponding to Table 3 and Table 4, and Table 3 are shown in the Figure 11. The results are as analyzed, the TC2NND algorithm has the best performance, and the detection performance is close to the TC2NND algorithm without applying TL technology and spending more time training.

In summary, the performance advantages of the TC2NND over traditional methods (such as cyclostationary detection) and the single-model C2NND approach can be explained by the following:

(1) The TC2NND divides the complex CPS feature space according to SNR levels, where each C2NND sub-model is responsible for classification within a specific SNR range (e.g., C2NND1 handles −19 to −15 dB). With residual connections, the TC2NND achieves an accuracy of 73.2% under low SNR conditions, as shown in Table 4. In contrast, the cyclostationary detection methods lack this adaptability (seen in Figure 10a). Also, the single-model C2NND tends to overfit high-SNR data, which leads to a 10% drop in detection probability at low SNRs, as demonstrated in the comparison between Figure 9c and Figure 10c.
(2) The traditional energy detection is limited by noise uncertainty, and cyclostationary detection relies on accurate selection of the cyclic frequency. The TC2NND addresses these issues by using an SNR classifier that puts 98% of noise samples into ${\mathrm{C2NND}}_{1}$ (seen in Figure 5), which is designed to handle classification under low SNR conditions. The single-model C2NND lacks this capability and fails to meet detection requirements under extremely low false-alarm rates.
(3) By applying transfer learning from high to low SNR, the TC2NND transfers robust features between models. This reduces training time for low SNR cases by 50% (Table 3 vs. Table 5), and improves detection probability by 12% at SNR = −15 dB (seen in Figure 11d). Therefore, the transfer learning helps overcome the limitation caused by insufficient low-SNR training data.

## 6. Conclusions

In conclusion, this paper has presented the TC2NND for maritime cognitive radio network spectrum sensing, which regards the spectrum sensing problem as a classification task. We use the FAM technique to extract the features of the signal and design a classification-guided multi-network TC2NND to solve the classification problem. Experimental results demonstrate that the TC2NND algorithm achieves a detection probability of 78% while maintaining a low false-alarm probability of 1% at an SNR of −10 dB. It is notable that this performance exceeds that of existing cyclostationary feature detection methods by 65%. This performance enhancement is particularly notable at low SNR regimes, where the TC2NND algorithm maintains a robust detection capability, ensuring reliable communication with minimal interference to primary users. Future work will consider datasets generated with a variety of modulation schemes, as well as the development of realistic datasets based on practical channel conditions. In addition, future research will explore more deep-learning models for spectrum sensing under multiple PU and SU, aiming to better reflect real-world scenarios and enhance applicability in distributed spectrum sensing environments.

## Figures and Tables

**Table 1:** Simulation parameters.

Simulation Parameters	Values
Modulation of PU	BPSK
Carrier frequency *f*	${\mathrm{1 \times 10}}^{6}$
Number of sampling points $L_{s}$	1000
The number of decision models *U*	3
SNR Interval (dB)	[−19, −15], [−14, −10], [−9, 10]
Number of signals per ${\mathrm{dataset}}_{u}$	2000
The total sample size of the data set *K*	12,000
Ratio of training set to validation set a:b	7:3

**Table 2:** Complexity comparison of methods.

	FLOPs	Average Processing Latency (ms)
Energy Detection	0.256 MFLOPs	0.1
Cyclostationary feature Detection	0.326 GFLOPs	1.47
C2NND Detection	8.19 GFLOPs	4.34
TC2NND Detection	16.41 GFLOPs	7.83

**Table 3:** The analysis of TC2NND.

	Training Time	Loss of Validset	Accuracy of Validset
${\mathrm{C2NND}}_{1}$	27.76 s	0.561	0.732
${\mathrm{C2NND}}_{2}$	34.37 s	0.264	0.934
${\mathrm{C2NND}}_{3}$	2 min 24.82 s	0.005	0.998

**Table 4:** The analysis of TC2NND without TL technology 1.

	Training Time	Loss of Validset	Accuracy of Validset
${\mathrm{C2NND}}_{1}$	34.19 s	1.151	0.520
${\mathrm{C2NND}}_{2}$	34.25 s	0.414	0.896
${\mathrm{C2NND}}_{3}$	2 min 26.36 s	0.005	0.998

**Table 5:** The analysis of TC2NND without TL technology 2.

	Training Time	Loss of Validset	Accuracy of Validset
${\mathrm{C2NND}}_{1}$	60.33 s	1.299	0.682
${\mathrm{C2NND}}_{2}$	66.13 s	0.269	0.918
${\mathrm{C2NND}}_{3}$	2 min 24.59 s	0.004	0.998

## Data Availability

The data that support the findings of this study are available from the corresponding author upon reasonable request.
